# Defining a Superlens Operating Regime for Imaging Fluorescent Molecules

**DOI:** 10.1371/journal.pone.0007963

**Published:** 2009-12-01

**Authors:** Kareem Elsayad, Katrin G. Heinze

**Affiliations:** Optical Engineering, Research Institute of Molecular Pathology (IMP), Vienna, Austria; Kings College London, United Kingdom

## Abstract

It has been shown that thin metal-based films can at certain frequencies act as planar near-field lenses for certain polarization components. A desirable property of such “lenses” is that they can also enhance and focus some large transverse spatial frequency components which contain sub-diffraction limit details. Over the last decade there has been much work in optimizing designs to reduce effects (such as material losses and surface roughness) that are detrimental to image reconstruction. One design that can reduce some of these undesirable effects, and which has received a fair amount of attention recently, is the stacked metal-dielectric superlens. Here we theoretically explore the imaging ability of such a design for the specific purpose of imaging a fluorescent dye (the common bio-marker GFP) in the vicinity of the superlens surface. Our calculations take into consideration the interaction (damping) of an oscillating electric dipole with the metallic layers in the superlens. We also assume a Gaussian frequency distribution spectrum for the dipole. We treat the metallic-alloy and dielectric-alloy layers separately using an appropriate effective medium theory. The transmission properties are evaluated via Transfer matrix (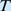-matrix) calculations that were performed in the MatLab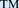 and MathCad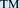 environments. Our study shows that it is in principle possible to image fluorescent molecules using a simple bilayer planar superlens. We find that optimal parameters for such a superlens occur when the peak dipole emission-frequency is slightly offset from the Surface Plasmon resonance frequency of the metal-dielectric interfaces. The best resolution is obtained when the fluorescent molecules are not too close (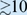 nm) or too far (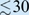 nm) from the superlens surface. The realization and application of a superlens with the specified design is possible using current nanofabrication techniques. When combined with e.g. a sub-wavelength grating structure (such as in the far-field superlens design previously proposed [1]) or a fast near-field scanning probe, it could provide a means for fast fluorescent imaging with sub-diffraction limit resolution.

## Introduction

In conventional far-field fluorescence microscopy the spatial resolution is severely restricted by the diffraction-limit [2]. Whilst there are ways to overcome this, current superresolution techniques are not well suited for fast dynamic wide-field studies. One potential approach which is not limited in this respect, would involve the use of so-called superlenses that preserve or enhance evanescent components of the electromagnetic field [3]. Planar superlens designs that operate at optical frequencies can consist of composite [4], [5] or stacked metallic films [6], [7], that in the electrostatic limit amplify and focus the Transverse Magnetic (TM) components of the field by exciting Surface Plasmon Polariton (SPP) resonances at the metal-dielectric interfaces [3]. Experimentally, setbacks have included surface roughness and material absorption [8]. However, advances in manufacturing ultra-smooth metallic films [9] offers incentive for studying such lenses in regard to specific imaging applications.

The use of superlenses for fluorescent imaging purposes is additionally complicated by the interactions between the fluorescent dyes and the metal film(s), which will distort the local field [10], [11] and may cause a significant change in the fluorescence decay-rate and intensity [1]–[7]. Superlenses where the anisotropy of a metal-dielectric stack is used as a type of waveguide for high spatial frequency components have more recently been proposed (e.g. the canalization [18] and resonant tunneling [7], [19] regimes). Since in these regimes the superlenses can operate slightly off resonance the performance, in regards to imaging fluorescent dye molecules, would be distinct from that in the conventional superlensing regime studied in e.g. [20]. In this article we present calculations for the ability of a stacked composite-metal/dielectric superlens operating off-resonance to image sources similar to the common fluorescent label Green Fluorescent Protein (GFP). We characterize the imaging capabilities using an analytic Transfer matrix (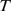-matrix) method often used for analyzing similar designs. By tuning the parameters of the superlens we find that there is a regime were the plasmonic amplification at the metal-dielectric surfaces together with the canalization-like effects from the anisotropy of the structure produce favorable conditions for superresolution imaging.

## Methods

The electric field of GFP can as a first approximation be taken as that of an oscillating electric dipole with a finite frequency spectrum, and described by a Gaussian with its peak at 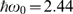 eV and a Full Width at Half Maximum (FWHM) of 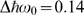 eV [21]. We can account for this by calculating the imaging ability of dipoles of all frequencies and integrating over frequencies with the appropriate Gaussian weighting factor.

**Figure 1:**
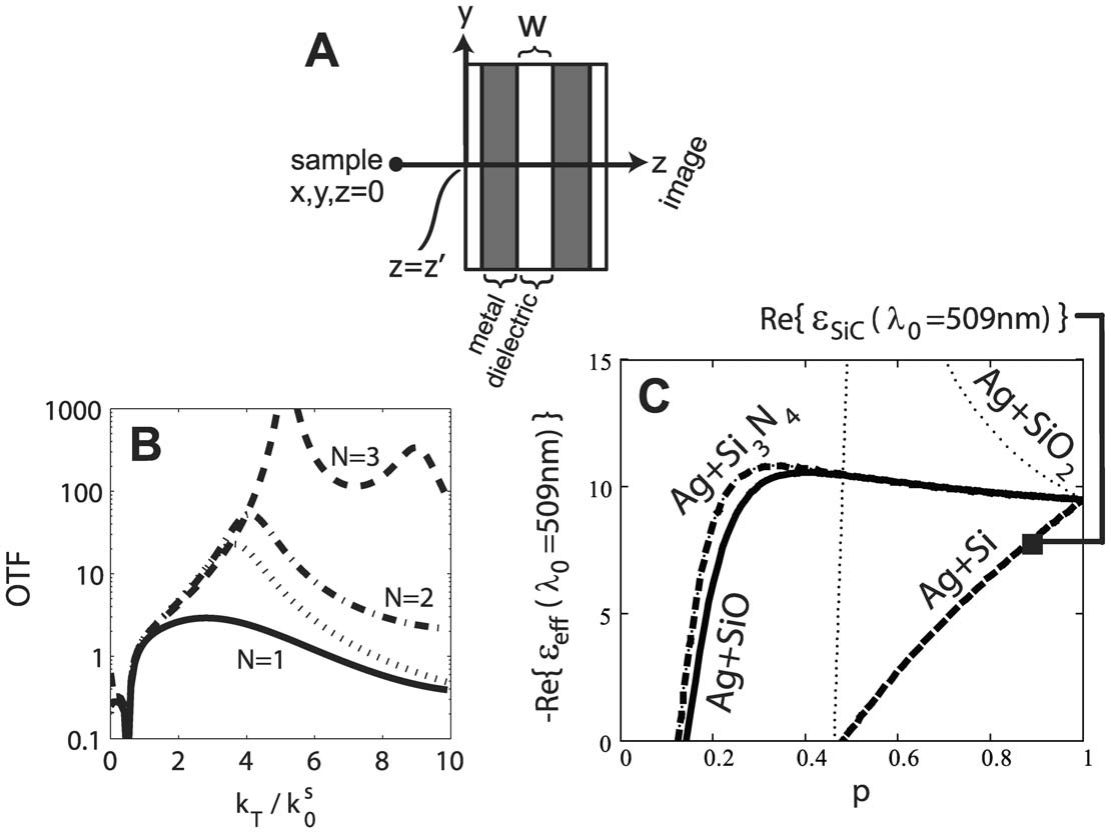
Superlens design and properties. (a) Sketch of the considered superlens with 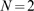. (b) The Optical Transfer Function (OTF) of the superlens as a function of 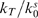 (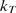 is the transverse wavevector and 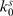 is the total wavevector in the source medium), for 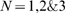. The total thickness and metallic-layer fraction is kept constant. The dotted line represents 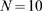. (**c**) Effective medium theory calculation of the real part of the dielectric function of the metal layers for various dielectric inclusions as a function of metal filling factor (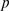). Values are evaluated at the effective emission frequency of a dipole (with 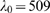 nm) a distance 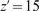 nm infront of the superlens (see text).

The superlens we consider consists of a stack of 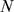 metallic layers and 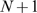 dielectric layers (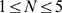), of thicknesses 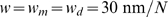, except for the two dielectric coating layers on the object and image face which are of thickness 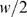. Fig. 1a shows a sketch of such a superlens with 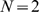. The medium on the source- and observer- side are chosen to be water (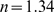) and air (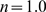). The sample (in our case an oscillating electric dipole) is located at 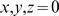, and the superlens front-face defines the 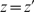 plane. The dielectric properties of the metallic layer can be tuned by mixing the metal with a dielectric [4]. The effective dielectric function thereby becomes 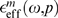, where 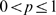 is the metal filling factor, and can be determined by Effective Medium Theory (EMT) and the frequency-dependent complex dielectric function of the constituent materials. Our calculations are based on a modified Maxwell-Garnett theory [22], [23] which has been shown to be as accurate as more elaborate numerical approaches used for high metal filling factors [24].

The interactions of electric dipoles with metallic surfaces and tips has been studied theoretically [12], [16], [17], [25] and experimentally [14], [15], [26] in various regimes for quite some time. The standing consesus is that, in the vicinity (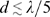) of continuous metallic films, the dipole decay-rate will increase with decreasing dipole-metal separations (typically as 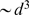). This is mainly due to the non-radiative coupling of the dipole to SPPs at the metal-dielectric interface, which provide an efficient non-radiative decay channel. As the dipole-metal distance is decreased further (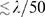) – below the Debye screening length – one has more efficient non-radiative coupling to SPPs, and a more rapid increase in the decay-rate (as 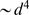). At these short distances, spatial dispersion effects need to be included explicitly, which can be done using the Random Phase Approximation (RPA) finite-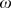 finite-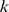 dielectric functions [27]. However, at such short distances the effects from surface roughness will also become more significant. Thus for applications where accurate localization of the SPPs is important (e.g. superlens imaging applications) it may not be desirable to operate at such distances.

Changes in the emission spectra and lifetime of a dipole due to near-field interactions with very thin (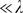) continuous [16] or discontinuous [15], [28], [29] metallic films or metamaterial structures [17, 3–2] can be quite different and less intuitive. Here we consider the effects that the superlens has on the emission frequency and the decay-rate of a dipole by treating the latter as a self damped harmonic oscillator. Using the model from [12] we then define an effective emission frequency 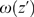 which is a function of dipole location 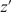 (see Fig. 1a). For our calculations we can assume the limiting cases 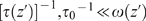, where 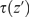 and 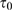 are the modified and un-modified life-times (inverse decay rates). In this limit both the decay-rate and the frequency-shift change approximately linearly with respect to the imaginary and real parts of the local field amplitudes respectively. These can be obtained using the 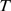-matrix formalism outlined below (in our case the metallic-dielectric layers can not be treated by an EMT since the dipole-superlens distance is comparable to the individual layer thicknesses). For parallel dipole orientations Transverse Electric (TE) polarization components will only become significant for higher frequencies. Thus, in calculating the modified decay-rates and frequency shifts we only consider the TM polarization components, which are chiefly responsible for the non-radiative coupling to the SPPs.

SPP resonances at the metal interfaces occur at 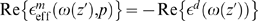 where 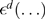 is the dielectric function of the dielectric layers. Requirements for an optimal metallic-layer include low absorption and a metal filling factor (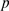) high enough above the percolation threshold. From a practical perspective it is also important that the metallic-alloy can be fabricated such that it is homogeneous on scales smaller than the desired imaging resolution. We find that out of several silver-alloys 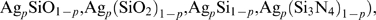 with different dielectric layers 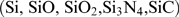, a combination that can satisfy these criteria is 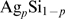 and SiC (see Fig. 1c). At 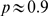, the resonance condition will occur for the GFP-dipole peak frequency (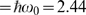 eV or 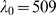 nm) located 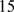 nm in front of the first layer. The parameters for this particular case are 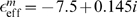 and 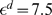. In practice fabrication technique and conditions will to a large extent determine the dielectric properties of metal alloys. Hence the applicability of a discrete Lorentz oscillator model for thin films is at best approximate [33], and the exact ratios will need to be determined semi-empirically. For all our calculations basic material parameters (e.g. plasma frequency and damping constant for Ag, and dielectric constants) are taken or extrapolated from [34].

To determine the incident field from the oscillating electric dipole we evaluate the complete electric dipole field [35] in Cartesian co-ordinates at 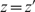 as a function of the angle that the dipole makes normal to the superlens-face. The contribution to the field from an arbitrary transverse wavevector component (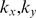) can then be picked out by taking the Fourier transform in the 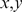 direction.

We write the electric fields with the position dependent modified frequency on the sample side of the superlens as 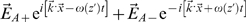, and that on the observer side as 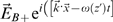. The components tangential to the superlens surface (in this case for the 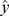-direction) are related by:
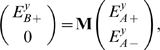
(1)where **M** is constructed from the interface transmission matrices (**T**) and propagation matrices (**P**). For a stacked superlens with 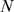 metal-dielectric layers, **M** is:
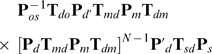
(2)


The indexes 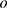, 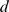, 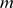 & 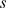 denote the air, dielectric, metallic and sample layers. The components of **P** for a layer 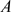 are given by: 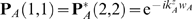, and 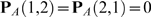, where 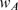 is the thickness of the layer. 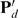 is the propagation matrix through the coating layer (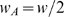), and 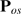 is the propagation matrix from the sample to the superlens-air interface. The components of **T** for the interface between layers 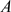 & 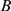 can be written as 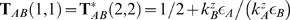, and 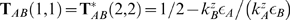. The values of 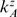 are determined by 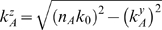, where 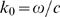 and 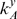 is obtained from the adjacent layer via the electric field boundary conditions.

The Optical Transfer Function (OTF) is determined by the ratio of the total emerging and incident field. For a range of superlens-source distances we find the largest OTF is obtained when 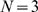 (see Fig. 1b for the 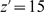 nm case). However, as one decreases the metal-layer thickness one increases the nearsightedness of the superlens, as there will no longer be sufficient distance for the “intermediate images” [3] to form. Decreasing the metal layer thickness also increases the percolation threshold and the conditions for the validity of the EMT used in calculating 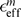. We thus focus on the 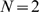 case. In doing so we have chosen a metal filling factor 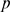 such that the dielectric-function mismatch is:
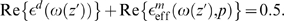
(3)In this way the canalization condition 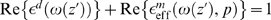 is not quite met, nor are we operating exactly on resonance. The canalization condition also requires 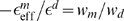, however the imaging ability is not too sensitive to slight adjustments in these parameters [36].

## Results and Discussion

**Figure 2:**
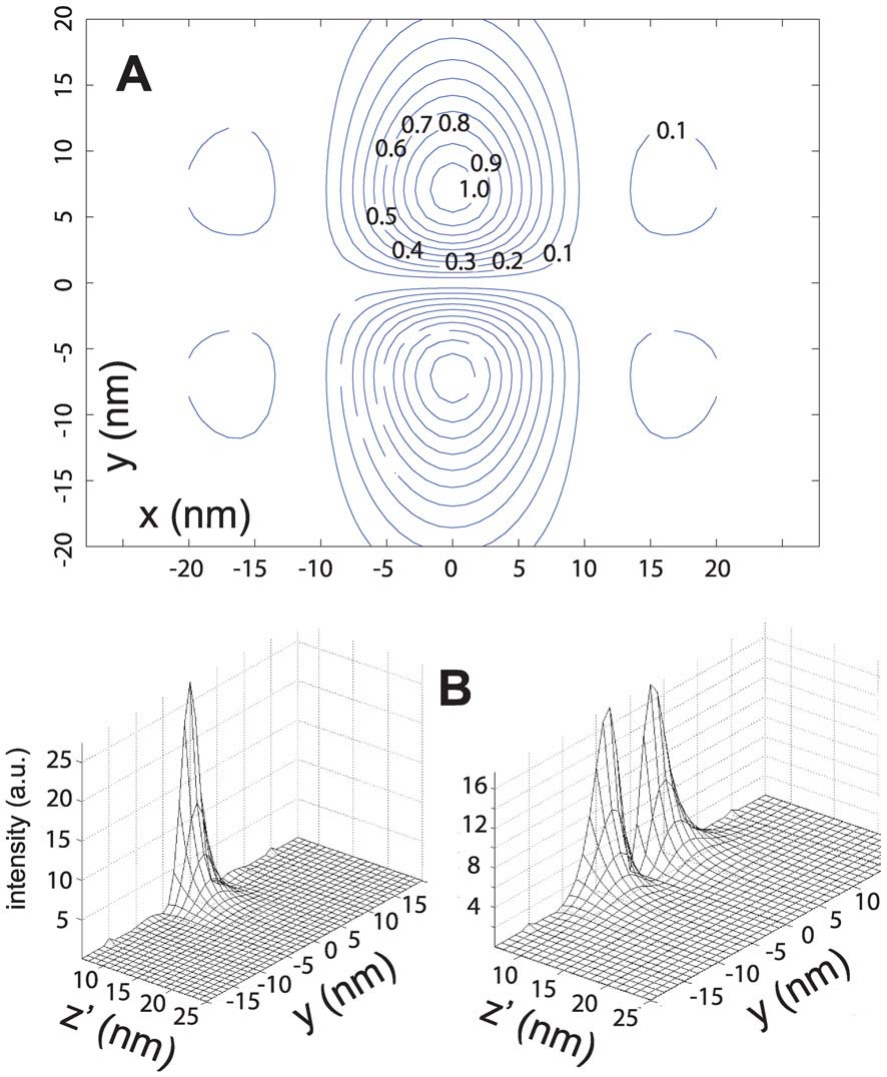
Field and intensity profiles of superlens images. (a) Contour plot of the 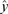-component of the electric-field amplitude (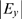, normalized to unity) in the image plane for a GFP-like source (dipole axis in 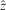-direction). Dipole is located at 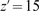 nm in front of the stacked superlens (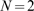) discussed in text. The metal filling factor “
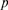
” has been adjusted so that Eqn. 3 is true for 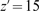 nm. (**b**) Electric field intensity for the superlens image of a GFP-like source when its dipole axis is parallel (in the 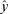-direction) [left] and perpendicular (in 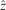-direction) [right]. The intensity profile of the image in the 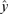 direction at 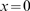 is plotted as a function of the source-superlens distance (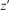). For each constant-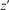 slice 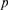 has been adjusted so that Eqn. 3 is true at the respective 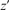.

Reconstructed images of oscillating electric-dipoles with their axis perpendicular and parallel to the superlens surface are studied as a function of distance from the superlens. We find that the transmitted intensity increases by a factor of 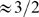 when the dipole axis is rotated from perpendicular (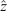) to parallel (for our case 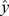). This is presented in Fig. 2b, where we show the total transmitted intensity through the superlens, for both dipole orientations, as a function of 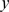 and superlens-dipole distance (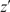) when 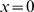. For each constant-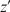 slice in this plot the parameter 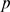 was adjusted such that Eqn. 3 is obeyed. The magnitude of the total transverse (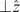) electric field in the image plane from a perpendicular (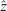) dipole would form an annulus (centered at 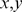 = 0), whereas that for a parallel (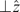) dipole would form an oval aligned parallel to the dipole-axis. In practice an average over dipole orientations will need to be taken; however here we focus on the case when the dipole axis is perpendicular (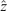) as this constitutes the ‘worse’ scenario in terms of incident intensity and thereby also localization ability. We note that if it is possible to measure particular in plane (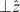) polarization components, as may be the case with the far-field superlens setup (see below), then the effective resolution may be further increased. This can be seen from Fig. 2a, where we show the magnitude of the 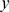-component of the electric field in the image plane for a 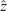-dipole at 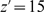 nm. By separately measuring the 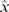 and 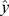 polarization contributions one could thereby localize the dipole to a higher precision in 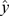 and 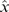 respectively.

**Figure 3:**
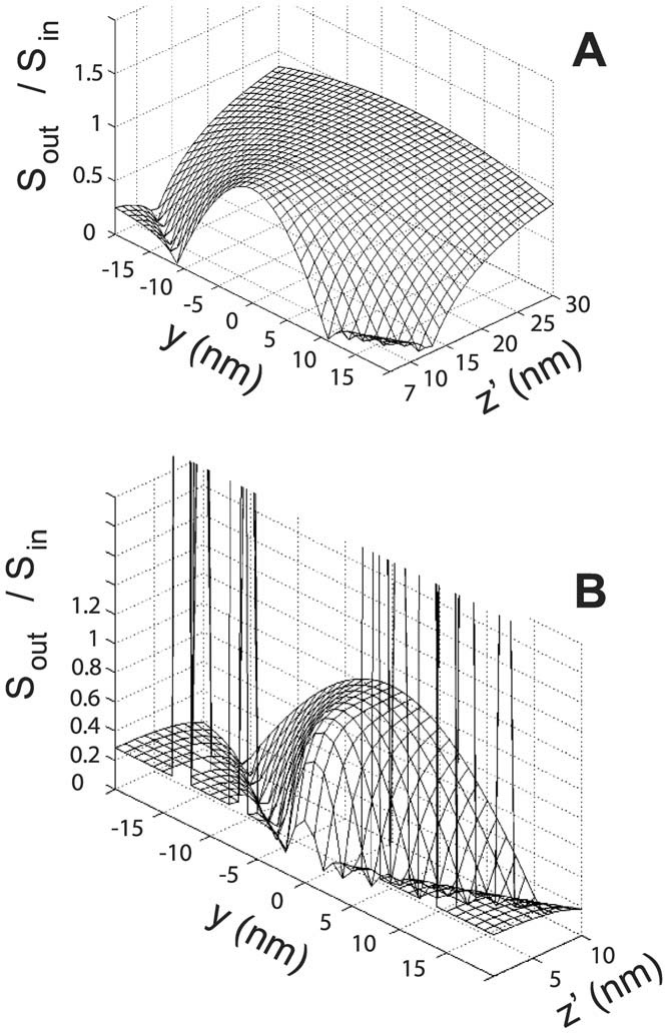
Transmission properties of an optimized-superlens. Fraction of transmitted energy as a function of “
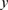
” and the dipole-superlens distance (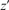). (**a**) 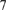 nm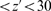 nm and (**b**) 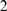 nm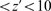 nm. “
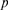
” has been chosen so that Eqn. 3 is true at 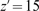 nm. All other parameters are as discussed in text.

We find that, for negligible absorption in both the source medium (water) and dielectric layer, the superlens is characterized by very high transmission in and around the 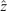 direction. In Fig. 3a & 3b we plot the ratio of the incoming and outgoing Poynting vector (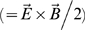), when the superlens is placed at various distances from a GFP-like source fixed at 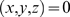. In these plots the parameter 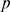 was *not* adjusted but fixed at 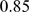, which corresponds to Eqn. 3 being true for 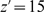 nm. Firstly we note that for dipole-lens distances smaller than 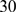 nm the transmission drops rapidly away from the 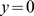 line (corresponding to a high resolution), and vanishes completely when 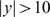 nm for superlens-dipole distances smaller than 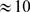 nm. As expected the range of very low transmission increases as the dipole-lens distance (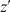) decreases. This occurs due to the (frequency-shifted) dipole emission peak moving further from the optimum operating frequency. Next we note that whilst the transmission peaks for 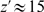 nm (not clearly visible in Fig. 3), the high transmission persists for distances below and above 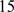 nm. Finally, it can be seen that as the source is moved closer to the superlens (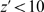 nm) singularities appear on either side of 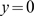, which correspond to propagating waves with large transverse components coupling to the SPPs. These singularities are also due to the layers becoming impedance matched (i.e. on resonance) at certain dipole-lens separations and for certain frequency components. We have found that if the superlens is made so that no impedance match occurs for any components of the frequency spectrum or dipole-lens distance, then the singularities are largely reduced. The surprising result that the optimum imaging of sources a finite distance from the superlens occurs when there is an impedance mismatch is not totally unexpected [18]. Here we have found that an optimized mismatched impedance design is also desirable for imaging dipole sources with finite-width frequency spectra interacting with the superlens.

**Figure 4:**
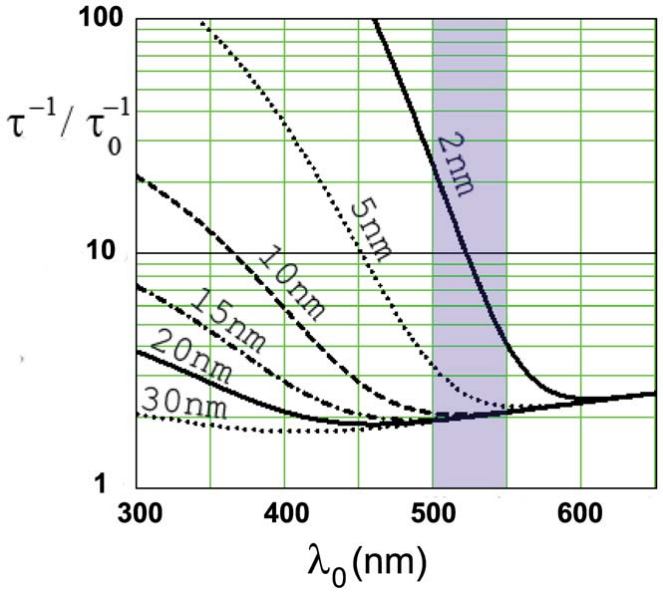
Decay-rate for an oscillating electric-dipole source in the vicinity of the proposed superlens as a function of dipole emission wavelength. Calculations are shown for 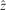-dipoles at several distances (*labeled on graph*) from the optimized superlens discussed in text. The highlighted (*blue*) band shows the transmission range for a typical band-pass filters used in GFP fluorescence imaging. 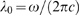, 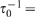
decay-rate for 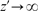.

**Figure 5:**
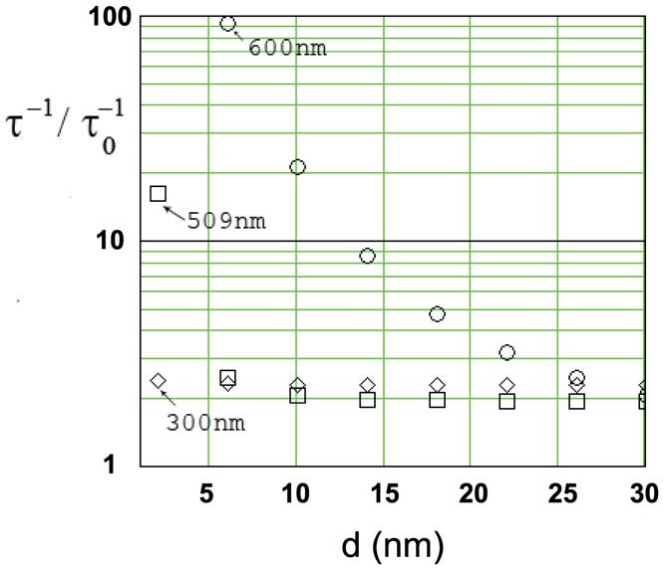
Decay-rate for an oscillating electric-dipole source as a function of dipole-superlens distance. Calculations are shown for three different emission wavelengths (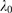, labeled on graph). Parameters are the same as Fig. 4.

The results of a similar self consistent calculation for the change in a 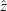-dipole decay rate as a function of superlens-dipole separation and dipole emission frequency are shown in Fig. 4 and Fig. 5 respectively. We see that for dipole-lens distances larger than 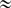
5 nm the decay-rate is modified only very little around the peak emission frequency, whereas at shorter wavelengths and distances it rapidly increases. This result may seem counter intuitive as one might expect the superlens to provide a highly effective non-radiative decay channel close to the resonance. However, our results support other recent calculations for anisotropic metal-dielectric slabs using the effective medium approximation where the decay-rate is found to be little affected in the vicinity of the SPP resonance [32]. Such features are likely to make the proposed structure particularly favorable.

In this manuscript we have theoretically studied a double-layer planar superlens for fluorescence imaging applications. We have found that when the parameters (geometrical and metal-filling factor) are tuned the superlens is characterized by a high transmission at desirable wavelengths. Whilst the maximum theoretical resolution of the proposed superlens may be very high (see e.g. Fig. 2 & Fig. 3), it will in practice be limited by the molecular brightness (photons emitted per molecule per second), the molecular stability, and noise. To quantify the imaging ability and usefulness of the proposed design we need to estimate the achievable resolution of a typical fluorescent marker.

A fluorophores brightness will depend in detail on numerous factors, such as the fluorophore's local environment [37], [38], the excitation intensity [39], and the particular mutation of the molecular complex [21]. For our calculations we consider a GFP molecule that emits an average of 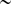
100,000 photons [39], with a lifetime (inverse decay-rate) of several nanoseconds [38]. Of these 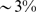 can be detected using available fluorescent imaging setups [39].

In the far-field the maximum resolution is often estimated using the Rayleigh Criterion. In the near-field however a definition of resolution is somewhat more arbitrary, and will depend a greater deal on the photon statistics [40]. If we include the contribution from quantum (shot) noise, the resolution 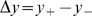 can then be estimated from:
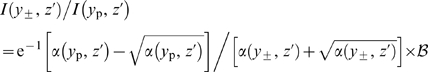
(4)for an image intensity profile 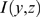 which peaks at 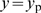. The photon statistics are accounted for in 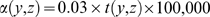, where 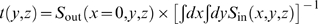 is the fraction of the total energy transmitted to a coordinate in the image plane. The fraction 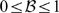 accounts for the additional effects from background and instrument noise, and is set to unity in our calculations. In practice 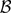 will always be smaller than unity and may as a first approximation be obtained from the signal-to-noise ratio (SN): 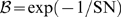. From Eqn. 4 and the image intensity profile from a GFP-like source located 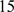 nm in front of the superlens with its dipole axis in the 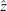-direction (Fig. 2b, right), we find a resolution of 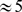 nm is achievable in the 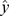-direction: by measuring the individual ‘lobes’. The resolution in the perpendicular (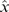) direction would by symmetry be the same. The same calculations for superlenses optimized for 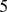 nm and 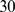 nm (by adjusting ‘
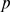
’ to satisfy Eqn. 3), give resolutions of 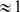 nm and 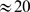 nm.

**Figure 6:**
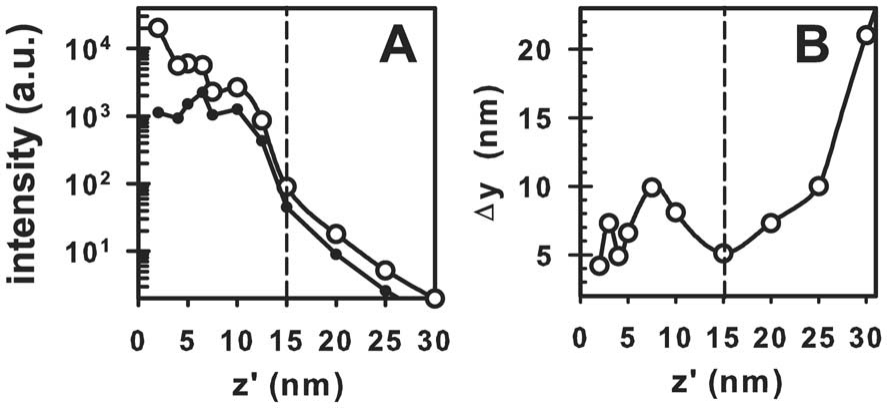
Imaging a GFP-like source (dipole axis in 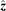-direction) with an optimized superlens. (a) Peak image intensity as a function of source-superlens distance 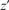, including (*open circles*) and neglecting (*solid circles*) modifications to the decay-rate from non-radiative coupling to the superlens. (b) Estimated resolution for the same setup. The dashed vertical line in both plots represents the distance at which Eqn. 3 holds. All superlens parameters are the same as for Fig. 3a & b.

In Fig. 6a & b we present the peak-intensity, and the maximum resolution for a superlens optimized for dipoles (dipole axis in 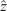) at 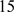 nm as a function of 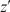. As can be seen from Fig. 6a the peak transmission occurs somewhat below 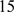 nm. This is due to the impedance match, which corresponds to in theory perfect transmission, occurring at a smaller superlens-dipole distance. The reason that the intensity effectively levels out is partly due to increased absorption in the metallic-layers (a closer dipole-lens distance implies a higher 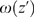, which gives a higher Im(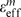)). The increased impedance mismatch that re-occurs will also decrease the transmission intensity. As the dipole gets closer than 15 nm the resonance condition is met and due to the singularities in the OTF causes a decrease in the resolution. We would also expect this from Fig. 3.

One interesting question is whether the change in the fluorescence decay-rate with fluorophore-superlens distance can be used for obtaining 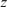-resolution. We find that this is theroretically possible, however practically unlikely: For the GFP emission-spectrum (highlighted region in Fig. 4) the decay-rate only changes appreciably at very short dipole-lens distances (Fig. 5) at which the imaging properties of the superlens are compromised (Fig. 3). Furthermore, at nanometer and sub-nanometer dipole-superlens distances strongly enhanced decay rates are predicted. Whilst, such fast decay rates could be supported by the superlens (plasmon lifetimes are ∼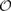 (10) fs [41]) it is unclear whether a GFP-like molecule would be able to undergo such rapid excitation-relaxation cycles.

A promising method of image acquisition would be to fabricate a sub-wavelength grating on the image side of the superlens. It is then possible to reconstruct the sub-diffraction limit details by measuring the scattered propagating negative diffraction orders in the far-field [1]. Unfortunately, such near-field to far-field scattering tricks, despite having long been known to the microwave community, are still in their infancy for optical imaging applications: the main bottleneck being limitations in precision nanoscale fabrication. In perspective of the rate of advancement of nanofabrication technologies, it is however likely that they will play an important role in fast near-field imaging in the not to distant future. A less favourable approach, however worth mentioning would be to read-out the image at the backface of the superlens using a Scanning Near-field Optical Microscopy (SNOM) probe. Whilst a SNOM approach would be eleborate and come with many of the pitfalls still associated with near-field scanning microscopy; the SNOM-superlens setup would have two important advantages over the SNOM only setup: (**1**) The complicated interactions of the sample with the dynamic SNOM probe [14] do not apply and are replaced by interactions of the sample with the static superlens. (**2**) The superlens-SNOM approach would allow for the study of processes in viscous or inhomogenious media and at (cell) surfaces in which direct SNOM scanning is either very complicated or not possible. In the SNOM-superlens setup the superlens may be fabricated on an ultra-thin silicon based membrane, which could in itself be part of the superlens structure - e.g. the middle dielectric layer (see Fig. 1).

In conclusion we have presented calculations that suggest the feasibility of imaging a GFP-like fluorescent source through a stacked superlens. We have proposed a design based on a double layer metal-alloy/dielectric stack that operates slightly off resonance. Experimental realization of the introduced superlens will rely on overcoming the undesirable effects of surface roughness and the challenges associated with image acquisition discussed above. However, recent advances in nanofabrication technologies give a bright outlook for the use of stacked superlenses, such as the one discussed in this manuscript, in fluorescence imaging applications.
